# Complete genome sequence of *Pedobacter* sp. strain NO84, a chain-like vesicle-producing bacterium

**DOI:** 10.1128/mra.00501-26

**Published:** 2026-10-15

**Authors:** Ayaka Makino, Hiromu Inoue, Akinori Soga, Wataru Kitagawa, Jun Kawamoto, Ryosuke Nakai

**Affiliations:** 1 Graduate School of Agriculture, Hokkaido University 12810, Sapporo, Japan; 2 Biomanufacturing Process Research Center, National Institute of Advanced Industrial Science and Technology (AIST), Sapporo, Japan; 3 Research Institute of Energy, Environment and Geology, Hokkaido Research Organization (HRO), Sapporo, Japan; 4 Institute for Chemical Research, Kyoto University, Uji, Japan; California State University San Marcos, San Marcos, California, USA

**Keywords:** genome analysis, Bacteroidota, membrane vesicle

## Abstract

We present the complete genome of *Pedobacter* sp. strain NO84, a chain-like vesicle-producing bacterium isolated from a 0.45-μm-filtered suspension of snow-covered soil. The genome comprises 4,125,118 bp with 3,585 protein-coding sequences. This genome provides a foundational resource for investigating vesiculogenesis and potential vesicle-based applications of this strain.

## ANNOUNCEMENT

Members of the genus *Pedobacter* (phylum *Bacteroidota*) attract industrial interest due to their production of enzymes used in therapeutics and aquaculture feed supplements (1, 2). We isolated a *Pedobacter* strain that produces extracellular chain-like membrane vesicles. Bacterial membrane vesicles can be genetically engineered to carry enzymes, providing platforms for extracellular biocatalysis (3). To facilitate future exploration of potential vesicle-based applications, we sequenced the whole genome of this strain.

**Fig 1:**
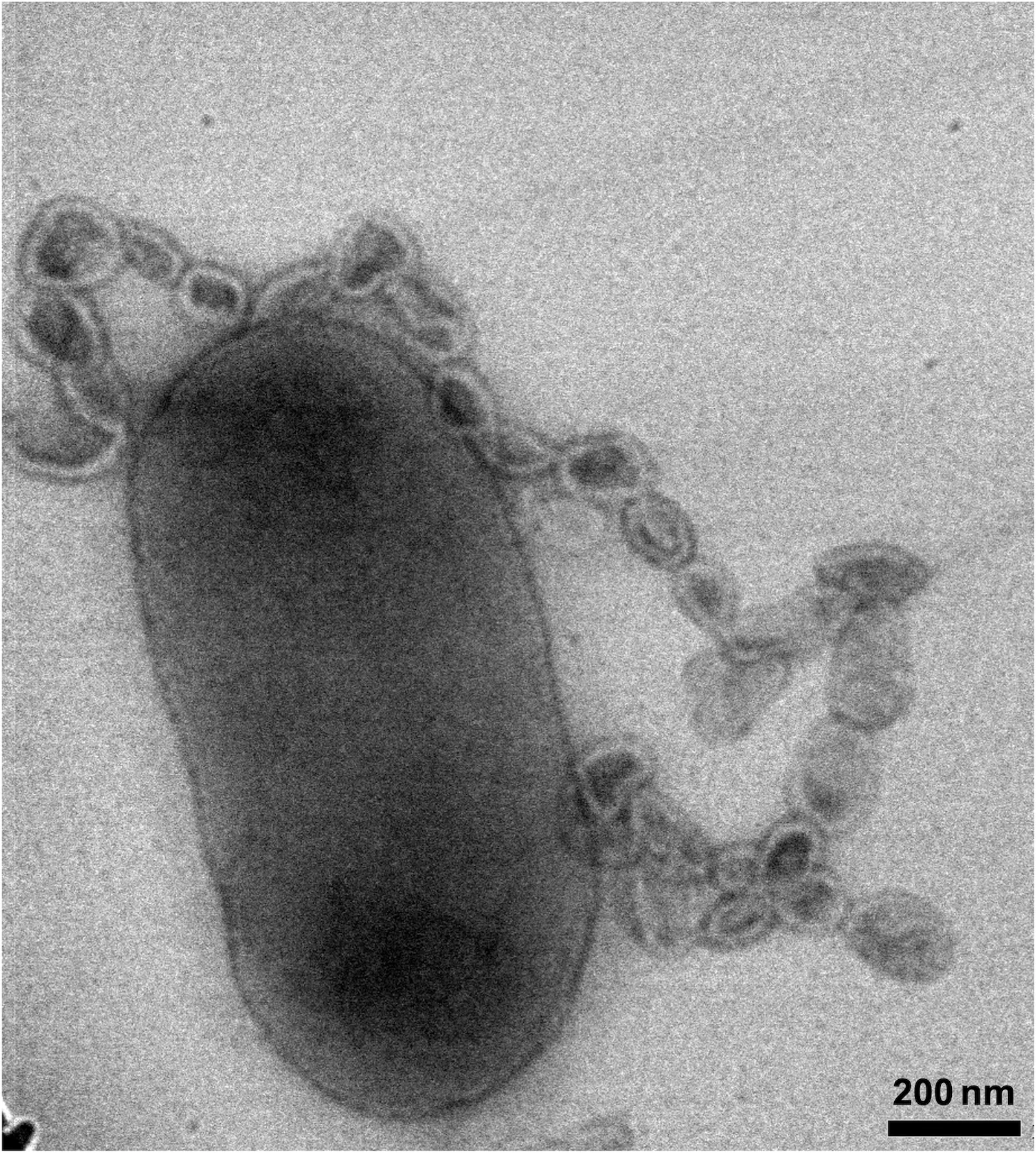
Transmission electron micrograph of *Pedobacter* sp. strain NO84. High-magnification image of chain-like vesicles secreted from the cell surface. Scale bar: 200 nm. Cells were grown statically in R2A medium at 25°C for 3 days and negatively stained with 1% uranyl acetate. The image was acquired using a JEM-1400 transmission electron microscope (JEOL, Ltd., Tokyo, Japan) at 120 kV.

Bacterial isolation began with a sample collected from snow-covered garden soil in Hokkaido, Japan (43.0201° N, 141.4182° E). A 3-g soil aliquot was suspended in 27 mL of ultrapure water, shaken (150 rpm, 30 min), and filtered through a sterile 0.45-µm syringe filter (Millex-HV; Merck Millipore, Burlington, MA) as described previously (4). The filtrate (50 µL) was plated onto 10-fold diluted Marine Broth 2216 agar medium (BD Difco, Franklin Lakes, NJ) and incubated at 20°C for 3 weeks. This isolation strategy, employing a micropore filter, targets novel filterable bacteria (5). From the isolates, 16S rRNA gene fragments were PCR-amplified using universal 27F and 1492R primers and then sequenced with the sequencing primer 907R, following the method described in reference 4. Cell morphology was also examined using phase-contrast and electron microscopy. Based on these data, strain NO84, a chain-like vesicle-producing *Pedobacter* bacterium, was selected for subsequent genome analysis (Fig. 1).

For genome sequencing, strain NO84 was cultured without shaking in R2A broth (DAIGO; Nihon Pharmaceutical, Japan) at 25°C for 2 weeks. DNA was extracted using a Genomic-tip 20G kit (QIAGEN, Valencia, CA). After DNA was sheared into approximately 10‒25 kb fragments with Megaruptor 3 (Diagenode, Seraing, Belgium), a sequencing library was prepared using a SMRTbell Prep Kit 3.0 and Revio Polymerase kit (Pacific Biosciences, Menlo Park, CA). The library was sequenced with Revio (Pacific Biosciences). The HiFi reads were obtained using SMRT Link ver. 13.1.0.221970 (Pacific Biosciences). Default settings were used for all software during data processing unless otherwise specified. PCR adapters, PCR duplicates, and short reads (≤1,000 bp) were removed using lima ver. 2.12.0 (https://github.com/pacificbiosciences/barcoding), pbmarkdup ver. 1.0.3 (https://github.com/PacificBiosciences/pbmarkdup), and Filtlong ver. 0.2.1 (with options “--min_length 1000” and “--keep_percent 90”) (6), respectively. This yielded 27,845 qualified HiFi reads with a mean length of 8,950 bp, totaling 249,217,187 bp. Reads were assembled with Flye ver. 2.9.3-b1797 using the “--pacbio-hifi” mode and option “-i 3” (7). Annotation was performed using DFAST ver. 1.3.6 (8). Genome completeness and contamination were estimated using CheckM (9) implemented in DFAST. The full-length 16S rRNA gene sequence from the genome was searched against the EzBioCloud 16S database (accessed August 2026) (10). Average nucleotide identity (ANI) was then calculated against the genome of the closest type strain using the EzBioCloud ANI Calculator (11).

The genome comprises a single circular chromosome of 4,125,118 bp with a 38.5% G + C content. The estimated genome completeness and contamination were 99.49% and 0%, respectively. The genome contains 3,626 genes, including 3,585 protein-coding genes, 6 rRNA genes, and 35 tRNA genes. The closest type strain to strain NO84 was *Pedobacter xixiisoli* CGMCC 1.12803^T^ (accession no. GCA_900215335.1), with 95.70% 16S rRNA gene sequence similarity and 72.29% ANI. Although the molecular basis of extracellular membrane vesicle production remains unelucidated, a curvature-sensing peptide approach was recently proposed to identify genes involved in this process (12). The genome sequence reported here serves as a foundational resource for future studies investigating chain-like vesicle production and its application.

## Data Availability

The NO84 genome sequence was deposited in the DDBJ/ENA/GenBank database under accession number AP046575 (BioProject PRJDB42040, BioSample SAMD01899374, and DDBJ Sequence Read Archive DRR959813).
